# The Chromomethylase CsCMT2 Negatively Affects Catechin Accumulation in Tea Plants

**DOI:** 10.3390/foods15203618

**Published:** 2026-10-11

**Authors:** Vita Tjandra, Zhan Yang, Yilin Yao, Xinxin Jia, Yang Zhang, Shifu Ma, Shujing Liu, Xinghui Li

**Affiliations:** 1 College of Horticulture, Nanjing Agricultural University, Nanjing 210095, China; vita_tjandra@yahoo.com (V.T.); 2024104080@stu.njau.edu.cn (Z.Y.); 13913120425@163.com (Y.Y.); 13602859717@163.com (X.J.); 2023204055@stu.njau.edu.cn (Y.Z.); 13993999795@139.com (S.M.); 2 Wenxian Tea Industry Development Service Center, Longnan 746400, China

**Keywords:** *Camellia sinensis*, chromomethylase, *CsCMT2*, DNA methylation, catechin biosynthesis, EGCG, tea flavor, tea quality, germplasm improvement

## Abstract

Catechins are the major bioactive polyphenolic compounds in tea (*Camellia sinensis*) and key determinants of its astringency, flavor quality, and health value. Although DNA methylation has been associated with catechin biosynthesis in tea, the roles of chromomethylase (CMT) genes in regulating catechin accumulation remain unclear. In this study, we characterized the CMT gene family in *C. sinensis* and identified four members: *CsCMT1*, *CsCMT2*, *CsCMT3a*, and *CsCMT3b*. All *CsCMT* genes were preferentially expressed in buds and young leaves, the tissues harvested for tea production, and *CsCMT2* showed the most pronounced cultivar-dependent expression under *Colletotrichum gloeosporioides* infection. Transient overexpression of *CsCMT2* in tea leaves decreased the contents of all six measured catechins (EGCG, GCG, EGC, GC, EC, and C), with EGCG showing the greatest reduction. It also downregulated seven catechin biosynthetic genes, namely *CsPAL*, *CsC4H*, *CsCHI*, *CsF3′5′H*, *CsDFR*, *CsANR*, and *CsSCPL1A*. Conversely, virus-induced gene silencing of *CsCMT2* increased total catechin content and upregulated these genes. Caffeine content was not significantly affected by either manipulation, indicating a relatively specific effect on catechin metabolism. These results suggest that *CsCMT2* negatively affects catechin accumulation associated with transcriptional changes in pathway genes and is a potential target for modulating catechin composition and flavor quality in tea germplasm improvement.

## 1. Introduction

Tea (*Camellia sinensis* (L.) O. Kuntze) is produced from the buds and tender shoots of the tea plant and is the most extensively consumed non-alcoholic beverage globally after water [1,2,3]. Its worldwide popularity rests on two attributes: a distinctive flavor and well-recognized health benefits. Both derive from a complex composition of carbohydrates, free amino acids, phenolic compounds, methylxanthine alkaloids, and volatile aroma compounds, which together give tea infusions antioxidant, anti-inflammatory, and other health-related activities [4,5,6,7]. Tea quality is determined by the balance among these metabolites [8], of which catechins, caffeine, and theanine are the main determinants [9]. Catechins contribute mainly to the astringency and part of the bitterness of tea infusions, caffeine contributes to bitterness [10], and theanine contributes to the umami taste [11,12]. Improving tea flavor and health value therefore depends to a large extent on understanding and controlling the accumulation of these compounds, particularly catechins.

Catechins are the most abundant polyphenols in tea and are central to both its taste and its health value. They comprise catechin (C), epicatechin (EC), gallocatechin (GC), and epigallocatechin (EGC), together with the galloylated forms epicatechin gallate (ECG), gallocatechin gallate (GCG), and epigallocatechin gallate (EGCG). EGCG is the predominant catechin in tea and accounts for much of the antioxidant and pharmacological activity of tea infusions [13,14,15,16]. Catechin content and composition differ markedly among cultivars and are further shaped by cultivation practices and environmental conditions [17]. Plucking season, shading, soil, altitude, light, temperature, atmospheric CO_2_, cultivar, and processing method all influence the metabolite profile and, consequently, the sensory and health attributes of the harvested leaf [18,19,20,21]. High-altitude cultivation, for example, has been reported to increase amino acid accumulation and lower catechin content, producing a mellower taste [22]. Within the plant, catechins accumulate most strongly in the bud and young leaves, the tender tissues that are harvested for tea production, and their content declines as leaves mature [23]. Because catechin content and composition largely determine the balance among astringency, bitterness, and mellowness, as well as the health value of tea, identifying the genes that control catechin accumulation is essential for the improvement of tea germplasm [8]. Although the biosynthetic pathway is now well defined, the mechanisms by which genetic factors regulate metabolic flux through this pathway remain incompletely understood [22].

Catechins are synthesized through the phenylpropanoid and flavonoid pathways [23,24]. Phenylalanine ammonia-lyase (PAL), cinnamate 4-hydroxylase (C4H), and 4-coumarate: CoA ligase produces p-coumaroyl-CoA, which chalcone synthase (CHS) and chalcone isomerase (CHI) convert into naringenin. Flavanone 3-hydroxylase (F3H), flavonoid 3′-hydroxylase, and flavonoid 3′,5′-hydroxylase (F3′5′H) then generate dihydroflavonols with different B-ring hydroxylation patterns, which dihydroflavonol 4-reductase (DFR) reduces to leucoanthocyanidins. Leucoanthocyanidin reductase (LAR) converts leucoanthocyanidins into the non-epimeric catechins C and GC, whereas anthocyanidin synthase and anthocyanidin reductase (ANR) produce the epimeric catechins EC and EGC. Finally, serine carboxypeptidase-like acyltransferase 1A (SCPL1A) catalyzes the galloylation that yields ECG and EGCG. The transcript levels of these structural genes are closely associated with catechin accumulation in tea leaves across seasons and growth conditions [25,26], and MYB transcription factors have been shown to regulate flavonoid biosynthesis in tea [27], indicating that transcriptional control is a major determinant of catechin content. In contrast, caffeine is synthesized through a separate purine alkaloid pathway, in which tea caffeine synthase 1 (TCS1) catalyzes the final methylation steps [28,29,30,31], and its expression is negatively regulated by the transcription factor CsS40 [32]. Theanine is likewise produced through an independent route involving theanine synthetase I (CsTSI) and alanine decarboxylase (CsAlaDC) [33,34,35,36,37]. Because these pathways are largely separate, a regulator acting on catechin biosynthesis could alter the astringency and health-related polyphenol content of tea without changing its caffeine content.

Beyond transcription factors, gene expression in plants is also regulated by epigenetic mechanisms, particularly DNA methylation [38,39]. Plant DNA methylation occurs in the CG, CHG, and CHH sequence contexts (where H = A, T, or C) and is maintained by different classes of DNA methyltransferases. Among them, the plant-specific chromomethylases (CMTs) are mainly responsible for methylation in the non-CG contexts [38,40]. CMT proteins contain a bromo-adjacent homology (BAH) domain and a chromodomain in addition to the catalytic methyltransferase domain, and these modules link CMT activity to histone H3 lysine 9 dimethylation [41]. Plant CMT genes are divided into three subfamilies, CMT1, CMT2, and CMT3 [42]. In *Arabidopsis thaliana*, CMT3 is required for the maintenance of CHG methylation [43], whereas CMT2 is required for CHH methylation in heterochromatic regions [44,45]. DNA methyltransferase genes, including CMT genes, have been analyzed at the genome-wide level in crops such as cotton and rubber tree [46,47]. In tea plants, CMT genes were identified as part of the cytosine-5 DNA methyltransferase family [48]. However, previous analyses of tea CMT genes focused mainly on their responses to abiotic stress and withering [48], whereas their expression under biotic stress and their roles in quality-related metabolism remain unclear.

Increasing evidence indicates that DNA methylation participates in the formation of tea quality. Integrated methylome, transcriptome, and metabolome analyses have shown that changes in the methylation of genes involved in flavor compound biosynthesis are associated with differences in metabolite accumulation among tissues and during oolong tea processing [49], as well as with seasonal changes in secondary metabolism [50]. During leaf development, global DNA methylation increases from tender to old leaves, and hypermethylation occurs mainly in genes of the phenylpropanoid, flavonoid, and terpenoid pathways. The effects of this methylation depend on its genomic location and sequence context, and changes in catechin content are consistent with changes in the methylation and expression of catechin biosynthetic genes such as *CsSCPL1A* and *CsLAR* [21]. These studies suggest that DNA methyltransferases, including chromomethylases responsible for non-CG methylation, may influence catechin biosynthesis. Catechin metabolism is also closely linked to biotic stress. Anthracnose, caused by *Colletotrichum* species including *C. gloeosporioides*, is one of the most damaging foliar diseases of tea, reducing both yield and quality [51], and catechin accumulation forms part of the defense response of tea plants against *C. gloeosporioides* [52]. In addition to biotic stress, environmental factors, including light spectrum, intensity, and photoperiod, influence tea specialized metabolite accumulation by activating photoreceptor and redox-dependent signaling that upregulates phenylpropanoid and flavonoid biosynthetic genes [53]. Nevertheless, the evidence linking DNA methylation to catechin biosynthesis in tea remains largely correlative, and the functional roles of chromomethylase genes in regulating catechin accumulation and tea quality remain unclear.

In this particular study, we performed a genome-wide characterization of the CMT gene family in *C. sinensis* and examined the expression of *CsCMT* genes in different tissues and in eight cultivars infected with *C. gloeosporioides*. *CsCMT2*, the sole member of the CMT2 subfamily in tea, exhibited the most pronounced cultivar-dependent expression variation under this biotic stress. Given that CMT2-type enzymes mainly mediate non-CG methylation and that catechin accumulation contributes to the defense response of tea plants against this pathogen, we hypothesized that CsCMT2 participates in the regulation of catechin biosynthesis. To test this hypothesis, *CsCMT2* was transiently overexpressed and silenced by virus-induced gene silencing (VIGS) in tea leaves, followed by the determination of catechin and caffeine contents and the expression analysis of catechin biosynthetic genes. This study suggests that CsCMT2 is a candidate negative regulator of catechin biosynthesis and a potential target for tailoring catechin composition and flavor quality in tea.

## 2. Materials and Methods

### 2.1. Plant Materials and Growth Conditions

Two-year-old tea seedlings (*C. sinensis* cv. ‘Shuchazao’) were grown in a growth chamber at the College of Horticulture, Nanjing Agricultural University (Nanjing, China), under controlled conditions (24 °C, 60–70% relative humidity (RH), and a 12 h light/12 h dark photoperiod). For each treatment, leaf samples were collected, immediately frozen in liquid nitrogen, and stored at −80 °C until RNA extraction and metabolite analysis. The replication structure of each experiment is described in Section 2.4, Section 2.6, Section 2.7, and Section 2.9.

### 2.2. Genome-Wide Identification of Chromomethylase (Cmt) Genes in Tea Plant

The *Arabidopsis thaliana* CMT protein sequences were retrieved from The Arabidopsis Information Resource (TAIR; https://www.arabidopsis.org) (accessed on 13 July 2026) and used as queries in BLASTP searches (E-value < 1.0 × 10^−5^) against the *C. sinensis* cv. Shuchazao chromosome-level genome (V2) from the Tea Plant Information Archive (TPIA; https://tpia.teaplants.cn) (accessed on 13 July 2026) [24] to obtain candidate *CsCMT* sequences. A single transcript is annotated for each CsCMT gene in this assembly. Physicochemical parameters of the deduced CsCMT proteins, including sequence length, molecular weight (MW), theoretical isoelectric point (pI), instability index, aliphatic index, and grand average of hydropathicity (GRAVY), and subcellular localization were predicted with ProtParam (https://www.expasy.org/) (accessed on 13 July 2026) and WoLF PSORT (https://wolfpsort.hgc.jp/), (accessed on 13 July 2026) respectively. Pairwise amino acid identity was calculated with DNAMAN 7.0. The presence of conserved domains in each CsCMT protein was verified using PfamScan (https://www.ebi.ac.uk/Tools/pfa/pfamscan/) (accessed on 13 July 2026).

### 2.3. Phylogenetic, Gene-Structure and Promoter Cis-Element Analyses

CMT protein sequences from *C. sinensis*, *A. thaliana*, *Actinidia eriantha*, *Cornus florida*, *Cynara cardunculus*, and *Diospyros lotus* were aligned in MEGA X [54] using MUSCLE with default settings. Phylogenetic relationships were inferred by neighbor-joining under the p-distance model with pairwise deletion of gaps, and node support was estimated from 1000 bootstrap replicates. Exon–intron organization was rendered with TBtools (version 1.x) [55] from the *C. sinensis* GFF3 annotation. Conserved motifs were detected with MEME v4.2.0 (maximum 20 motifs; otherwise, default) [56]. Promoter regions spanning 2000 bp upstream of the translation start site of each *CsCMT* gene were obtained from the *C. sinensis* genome and screened for *cis*-regulatory elements using PlantCARE [57].

### 2.4. Expression Profiling of Cscmt Genes

To validate the expression profiles and examine the spatial expression of *CsCMT* genes, selected members (*CsCMT1*, *CsCMT2*, *CsCMT3a/b*) were analyzed by quantitative real-time PCR (RT-qPCR). Samples were collected from different tissues and organs (flower, stem, root, apical bud, young leaf, mature leaf, and old leaf) to assess tissue-specific expression patterns. In addition, expression of the three *CsCMT* genes was examined under biotic stress in eight different tea cultivars (‘Shuchazao’, ‘Chuyeqi’, ‘Longjing 43’, ‘Baiye1’, ‘Zhongcha108’, ‘Jinmudan’, ‘Jinxuan’ and ‘Xinyang10’) caused by infection with the anthracnose pathogen *Colletotrichum gloeosporioides*. For each tissue and cultivar, three independent biological replicates were collected.

### 2.5. Rna Extraction and Rt-Qpcr

Total RNA was extracted using the RNAprep Pure Plant Plus Kit (TIANGEN, Beijing, China) as per the protocols outlined by the manufacturer. A total of 1 microgram (μg) of total RNA per sample served as a template for first-strand cDNA synthesis with the HiScript II 1^st^ Strand cDNA Synthesis Kit (Vazyme, Nanjing, China). Quantitative real-time PCR (RT-qPCR) was executed on an ABI QuantStudio 6 Flex instrument (Thermo Fisher Scientific, Waltham, MA, USA) using AceQ Universal SYBR qPCR Master Mix (Vazyme, Nanjing, China). Relative gene expression was then assessed by the 2^−ΔΔCT^ method [58] using *CsGAPDH* and *CsACTIN* as internal reference genes. All primer sequences employed in this study are provided in Table S5.

### 2.6. Transient Overexpression of Cscmt2

The coding sequence (CDS) of *CsCMT2* was amplified from *C. sinensis* cv. ‘Shuchazao’ cDNA, verified by 1.2% agarose gel electrophoresis, transformed into Escherichia coli DH5α, and confirmed by Sanger sequencing (GenScript, Nanjing, China). The verified CDS was inserted into the pCAMBIA1301 vector, which harbors a *β-glucuronidase* (GUS) reporter cassette, and the resulting construct was introduced into *Agrobacterium tumefaciens* strain GV3101. Agrobacterium cultures were prepared as described in Section 2.7, and the infiltration suspension was injected through the petiole into the leaves of shoots at the three to four-leaf stage. After infiltration, the plants were kept in the dark at 24 °C for 24 h and then returned to the growth conditions described in Section 2.1. Each construct was infiltrated into three independent tea seedlings, which served as the three biological replicates. For each biological replicate, three infiltrated leaves were harvested and analyzed as technical replicates. Control treatments are described in Section 2.7, and primers are listed in Table S5.

### 2.7. Vigs-Mediated Silencing of Cscmt2

A 254 bp *CsCMT2*-specific fragment was amplified from *C. sinensis* cv. ‘Shuchazao’ cDNA and inserted into the pTRV2 vector to generate pTRV2-*CsCMT2*, following the VIGS system established for tea plants [59]. The recombinant construct was confirmed by Sanger sequencing, and the fragment sequence is provided in Supplementary Table S7. The pTRV1, pTRV2 (empty), and pTRV2-*CsCMT2* plasmids were separately introduced into *A. tumefaciens* GV3101. Agrobacterium cultures were grown at 28 °C with initial shaking at a speed of 200 rpm in LB medium supplemented with kanamycin (50 mg L^−1^) and rifampicin (25 mg L^−1^) until the OD_600_ reached 1.0, collected by centrifugation, and resuspended in infiltration buffer (10 mM MES, 10 mM MgCl_2_, and 200 μM acetosyringone). pTRV1 was mixed at a 1:1 ratio with either pTRV2 or pTRV2-CsCMT2, and 1 mL of the mixed suspension was injected through the petiole into shoots at the three to four-leaf stage. The plants were placed in the dark at 24 °C for 24 h and then returned to standard growth conditions, and the infiltrated leaves were collected after post-infiltration. Each construct was infiltrated into three independent tea seedlings, which served as the three biological replicates. For each biological replicate, three infiltrated leaves were harvested and analyzed as technical replicates. Primers used in this biochemical assay are listed in Table S5.

For both assays, leaves were infiltrated with the corresponding untreated wild-type (WT) leaves, empty vector pCAMBIA1301 without an insert for overexpression, and pTRV1 co-infiltrated with empty pTRV2 for silencing, which served as controls for wounding.

### 2.8. Determination of Catechins and Caffeine

Catechins and caffeine were extracted following the protocol of Tai et al. [60] with minor modifications adapted from Han et al. [50]. Freeze-dried powder (0.1 g) was mixed with 4 mL of aqueous methanol (80%, *v*/*v*), sonicated (30 min, 4 °C), centrifuged at 13,000 rpm for 10 min, and the supernatant was filtered through a 0.22 μm membrane filter. The extract was injected onto a Waters e2695 High-Performance Liquid Chromatography (HPLC) system (Waters Corporation, Milford, MA, USA) fitted with a 2489 UV/Vis detector and a Phenomenex C18 reverse-phase column (250 × 4.6 mm, 5 μm particle size). The mobile phases consisted of 0.2% (*v*/*v*) aqueous acetic acid (CH3COOH) (A) and methanol (CH3OH) (B), delivered by gradient elution, and peaks were identified against authentic standards. A representative chromatogram is shown in Supplementary Figure S3. Total catechin content was calculated as the sum of the six quantified catechins (EGCG, GCG, EGC, GC, EC, and C). The relative composition was expressed as the percentage of each catechin in the total catechin content. Fold change was calculated as the mean content of each catechin in a transgenic or silenced line divided by that of the corresponding vector control.

### 2.9. Statistical Analysis

Statistical analyses were carried out using IBM SPSS Statistics 27.0 (IBM Corp., Armonk, NY, USA). All comparisons, including gene expression across tissues and cultivars, as well as the metabolite and functional validation assays comparing wild-type (WT), empty-vector/pTRV2 controls, and treatment samples, were performed using one-way analysis of variance (ANOVA) followed by Tukey’s honestly significant difference (HSD) test. Statistical significance is indicated as * *p* < 0.05, ** *p* < 0.01, *** *p* < 0.001, and **** *p* < 0.0001, as specified in the figure legends.

## 3. Results

### 3.1. Genome-Wide Identification and Characterization of the Cscmt Gene Family in C. sinensis

BLASTP searches of the *C. sinensis* genome with the *A. thaliana* CMT proteins as queries recovered four family members, designated *CsCMT1* (CSS0013017), *CsCMT2* (CSS0003392), *CsCMT3a* (CSS0038763) and *CsCMT3b* (CSS0034027) according to their phylogenetic relationships with the *A. thaliana* CMTs (Table S1). The encoded proteins ranged from 624 to 845 amino acids (aas) (70.2–95.6 kDa) and were all predicted to be nuclear and hydrophilic (negative GRAVY values), consistent with their function as DNA methyltransferases. Three members were acidic (pI 4.95–6.01), whereas CsCMT2 was the only basic member (pI 8.29); CsCMT2 was also the shortest protein (624 aa), and CsCMT3a was the longest (845 aa).

**Figure 1:**
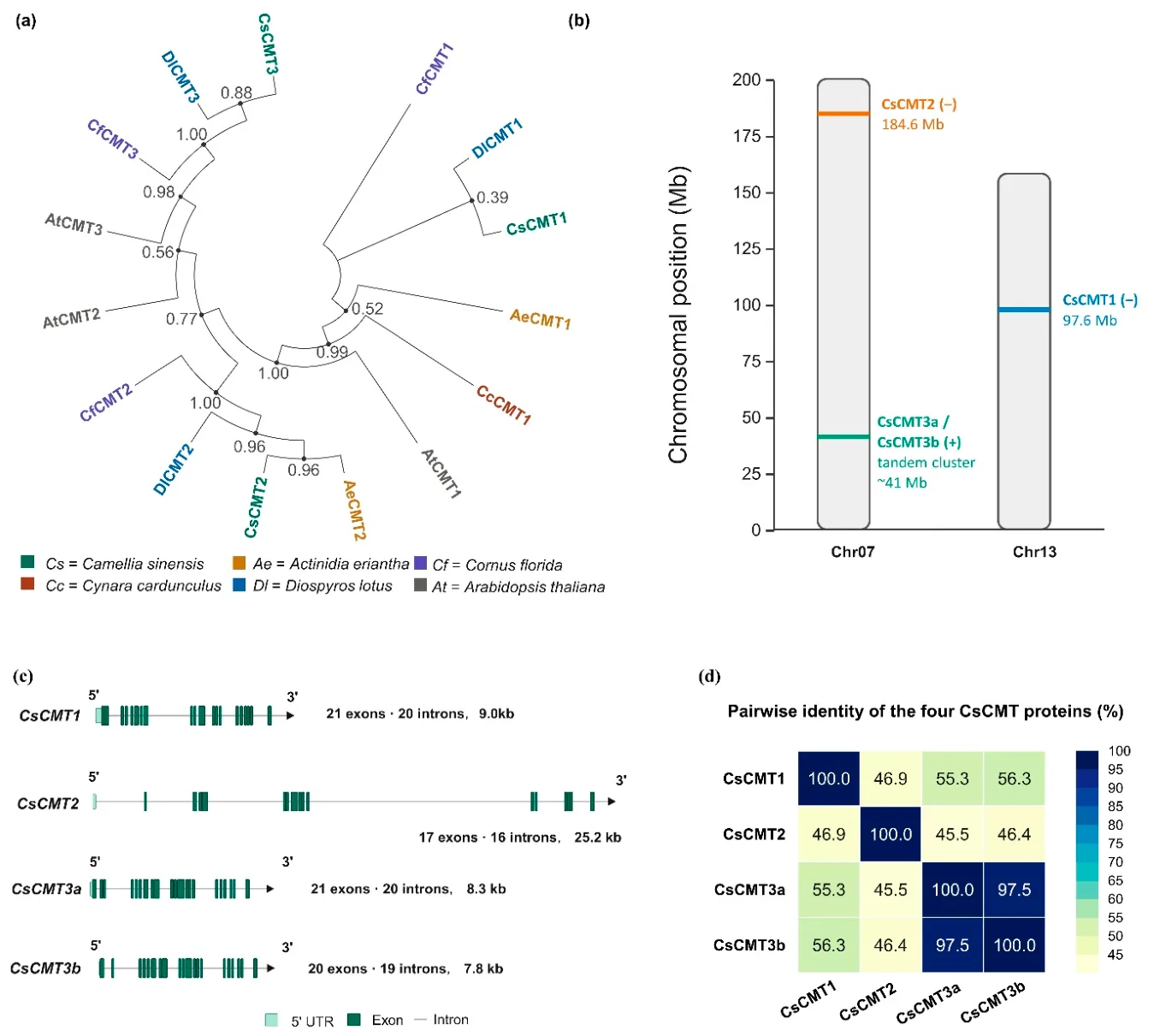
Genome-wide characterization of the CMT family in *Camellia sinensis*. (**a**) Neighbor-joining phylogenetic tree of CMT proteins from six species (*C. sinensis*, *A. thaliana*, *A. eriantha*, *C. florida*, *C. cardunculus*, and *D. lotus*). (**b**) Chromosomal locations of the *CsCMT* genes. (**c**) Exon–intron structures of the four *CsCMT* genes. (**d**) Heat map of pairwise amino acid identity (%) among the four CsCMT proteins.

A neighbor-joining phylogeny of CMT proteins from *C. sinensis*, *A. thaliana*, *A. eriantha*, *C. florida*, *C. cardunculus* and *D. lotus* resolved three subfamilies (Figure 1a). The CMT2 clade (CsCMT2, AeCMT2, DlCMT2, CfCMT2) and CMT3 clade (CsCMT3a, CsCMT3b, DlCMT3, CfCMT3, AtCMT3) were strongly supported (bootstrap 0.96–1.00 and 0.88–1.00, respectively), with CsCMT2 and AeCMT2 forming a sister pair. CMT1 was not monophyletic across the species sampled. Consistent with these assignments, the two *CsCMT3* paralogues formed a tight pair, while *CsCMT2* was the most divergent of the tea CMTs (Figure 1d, Table S2).

Pairwise identity analysis (Figure 1d and Figure S2) mirrored these relationships. CsCMT3a and CsCMT3b were near-identical (97.5% identity over the full-length alignment; identical across all 824 shared residues, with CsCMT3a carrying a 21-aa insertion), whereas identity between subfamilies was much lower (45.5–56.3%). CsCMT2 was the most divergent member, sharing only 45.5–46.9% identity with the other three proteins. The four genes were distributed on two chromosomes (Figure 1b). Three lay on chromosome 7, *CsCMT2* at 184.6 Mb and the *CsCMT3a/CsCMT3b* pair clustered near 41 Mb, while CsCMT1 was the sole member on chromosome 13 (97.6 Mb). *CsCMT3a* and *CsCMT3b* were arranged head-to-tail on the plus strand, 43 kb apart (Figure 1b), an arrangement consistent with tandem duplication. The exon–intron architecture also distinguished CsCMT2 (Figure 1c); *CsCMT1*, *CsCMT3a,* and *CsCMT3b* were compact, with 20–21 exons within 7.8–9.0 kb, whereas *CsCMT2* spanned 25.2 kb with the fewest exons (17) despite encoding the shortest protein. *CsCMT3a* carried one exon more than *CsCMT3b*.

All four proteins retained the chromomethylase-defining catalytic domain and chromodomain (Figure S1) but differed in N-terminal architecture. The region preceding the catalytic domain spanned 210–268 aa in CsCMT1, CsCMT3a, and CsCMT3b, sufficient to accommodate a BAH domain, but only 56 aa in CsCMT2, and the BAH domain was not detected in this CsCMT2 protein model.

Promoter analysis identified 20 types of *cis*-elements, which were categorized into four functional classes: light responsiveness, hormone signaling, stress response, and growth and development (Table S4). The *CsCMT1* promoter carried the largest and the *CsCMT2* promoter the smallest complement of elements, and *CsCMT3a* and *CsCMT3b* shared identical profiles, consistent with their duplication.

Across these analyses, *CsCMT3a* and *CsCMT3b* were distinguished only by two in-frame indels and by intron length, while *CsCMT2* differed from the other three members in isoelectric point, protein length, exon number, locus size, and N-terminal architecture.

### 3.2. Expression Patterns of Cscmt Genes in Different Tissues and in Cultivars Infected with C. gloeosporioides

**Figure 2:**
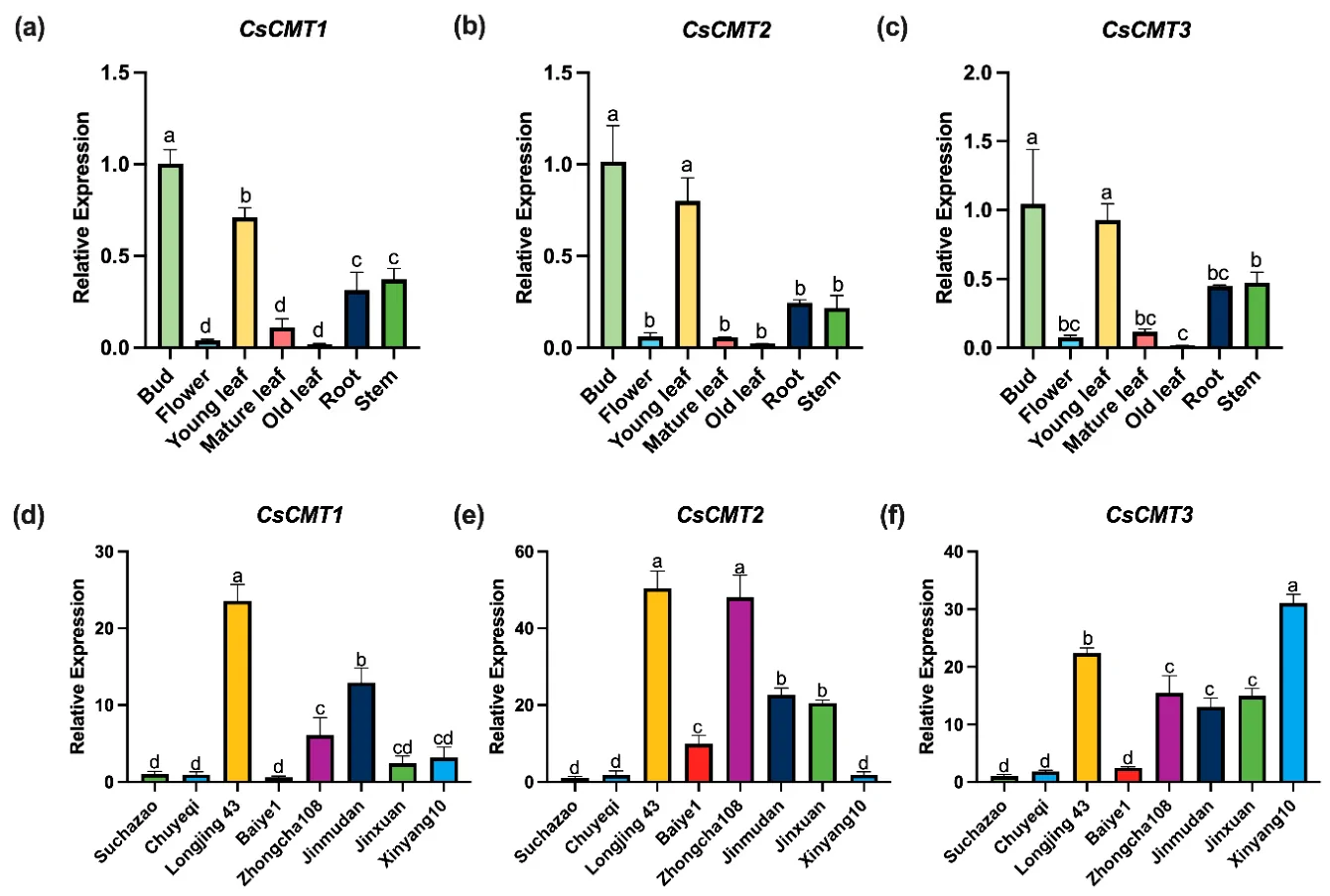
Expression of *CsCMT* genes across tea tissues and cultivars. (**a**–**c**) Relative expression of *CsCMT1*, *CsCMT2* and *CsCMT3* in seven tissues (bud, flower, young leaf, mature leaf, old leaf, root and stem). (**d**–**f**) Relative expression of the three genes in *Colletotrichum gloeosporioides*-infected tissue across eight tea cultivars (Shuchazao, Chuyeqi, Longjing 43, Baiye1, Zhongcha108, Jinmudan, Jinxuan and Xinyang10). Data are presented as mean ± SD (*n* = 3); different lowercase letters above the bars indicate significant differences among groups (Tukey’s HSD, *p* < 0.05).

The expression of the *CsCMT* genes was profiled across different tissues and under biotic stress in eight different tea cultivars caused by *Colletotrichum gloeosporioides* infection. Because *CsCMT3a* and *CsCMT3b* are nearly identical in coding sequence, encoding identical proteins over their shared length, they cannot be reliably distinguished by the transcriptome data and were therefore quantified together as a single CsCMT3. Expression is accordingly reported for three genes: *CsCMT1*, *CsCMT2 and CsCMT3*. Across the seven tissues examined, the three *CsCMT* genes shared a consistent, development-linked pattern; all were most strongly expressed in bud and young leaf and lowest in flower, mature leaf and old leaf, with root and stem at intermediate levels (Figure 2a–c). For *CsCMT1*, expression was highest in bud and young leaf, significantly lower in flower, mature leaf, and old leaf, and intermediate in root and stem (Figure 2a). *CsCMT2* followed the same profile, with bud and young leaf forming the top group and all remaining tissues significantly lower (Figure 2b). *CsCMT3* again peaked in bud and young leaf, with stem marginally above the other low-expressing tissues (Figure 2c). The progressive decline from young to mature and old leaf in all three genes indicates that *CsCMT* expression is associated with young, actively dividing tissues.

To compare *CsCMT* expression among cultivars under anthracnose infection, transcriptional expression levels of the three genes were measured by RT-qPCR in *C. gloeosporioides*-infected tissue of eight tea cultivars (‘Shuchazao’, ‘Chuyeqi’, ‘Longjing 43’, ‘Baiye1’, ‘Zhongcha108’, ‘Jinmudan’, ‘Jinxuan’ and ‘Xinyang10’). *CsCMT1* was highest in Longjing 43, followed by Jinmudan and Zhongcha108, and was low in the remaining cultivars (Figure 2d). *CsCMT3* was highest in Xinyang10 and Longjing 43 and lowest in Shuchazao, Chuyeqi and Baiye1 (Figure 2f).

Among the three genes, *CsCMT2* showed the most pronounced genotype-dependent expression under infection, varying more strongly among cultivars than *CsCMT1* or *CsCMT3* (Figure 2e). Together with its phylogenetic position as the sole tea member of the CMT2 clade, which is associated with the CHH methylation context previously correlated with catechin content, this result identified *CsCMT2* as the *CsCMT* member most likely to be involved in catechin metabolism. *CsCMT2* was therefore selected for functional analysis.

### 3.3. Cscmt2 Negatively Affects Catechin Accumulation

**Figure 3:**
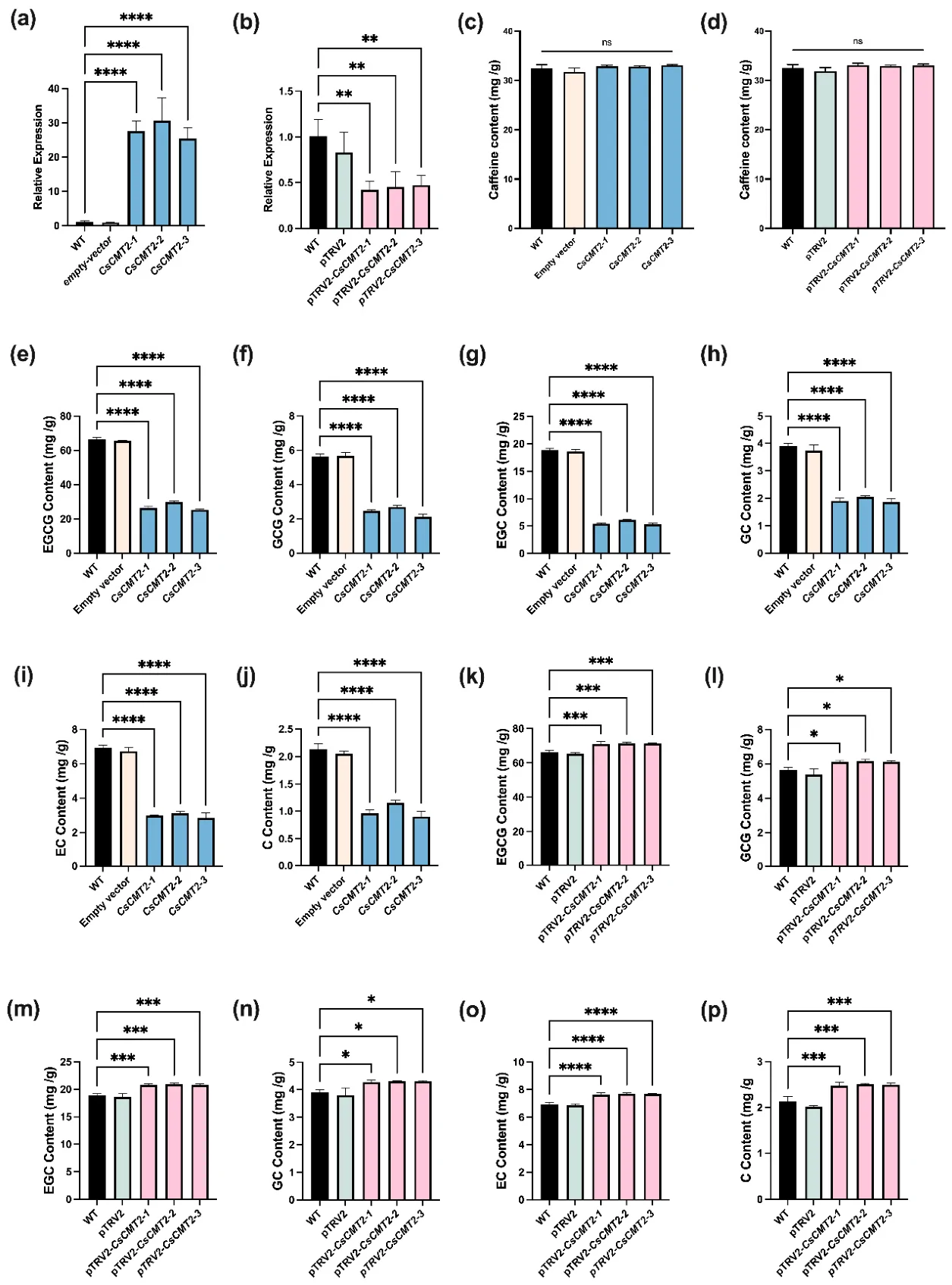
Effect of *CsCMT2* overexpression and silencing on catechin and caffeine accumulation in tea leaves. (**a**,**b**). RT-qPCR validation of *CsCMT2* transcript under overexpression and VIGS. (**c**,**d**). Caffeine content under overexpression and VIGS. (**e**–**j**). EGCG, GCG, EGC, GC, EC, and C in overexpression leaves. (**k**–**p**): the same six catechins in VIGS leaves. Controls: untreated wild-type (WT) and empty-vector and pTRV2 leaves (without CMT2 gene insert). Data are presented as mean ± SD (*n* = 3). Asterisks indicate significant differences compared with the wild type (WT) (one-way ANOVA with Tukey’s HSD; * *p* < 0.05, ** *p*< 0.01, *** *p* < 0.001, **** *p* < 0.0001).

To test the function of *CsCMT2* in tea secondary metabolites, we transiently overexpressed and silenced *CsCMT2* in tea leaves. RT-qPCR confirmed that both manipulations were effective, with *CsCMT2* transcript levels significantly increased in overexpression leaves and reduced in VIGS leaves compared with the empty-vector controls (Figure 3a,b). However, neither manipulation produced a visible change in leaf color (Figure S2), suggesting that CsCMT2 acts as an epigenetic regulator rather than a structural pigment gene. Empty-vector and pTRV2 controls did not differ from untreated wild-type (WT) for any metabolite measured, confirming that infiltration and TRV infection alone did not alter secondary metabolism. To distinguish changes in the concentration of individual catechins from changes in the size of the total catechin pool, we report the total catechin content, the relative composition of the pool, and the fold change in each catechin relative to its vector control (Table S6). Overexpression reduced all six catechins (Figure 3e–j). Total catechins decreased from 102.18 mg/g DW in the empty-vector control to 38.30–44.93 mg/g DW (0.37–0.44-fold; Table S6). Among individual catechins, EGC showed the largest relative decrease (0.29–0.33-fold), its share of the pool falling from 18.2% to 13.5–13.8%, whereas EGCG accounted for 62% of the absolute loss. Silencing (VIGS) produced the opposite effect (Figure 3k–p): total catechins increased from 102.02 to 112.40–112.98 mg/g DW (1.10–1.11-fold), with individual catechins increasing by 1.09–1.24-fold (Table S6). In contrast, caffeine content was not significantly affected by either manipulation (Figure 3c,d), suggesting that *CsCMT2* is more specifically associated with catechin metabolism than with secondary metabolism in general. Together, these reciprocal gain- and loss-of-function results identify *CsCMT2* as a candidate negative regulator of catechin accumulation in tea leaves.

### 3.4. Cscmt2 Modulates the Expression of Catechin Biosynthetic Genes

**Figure 4:**
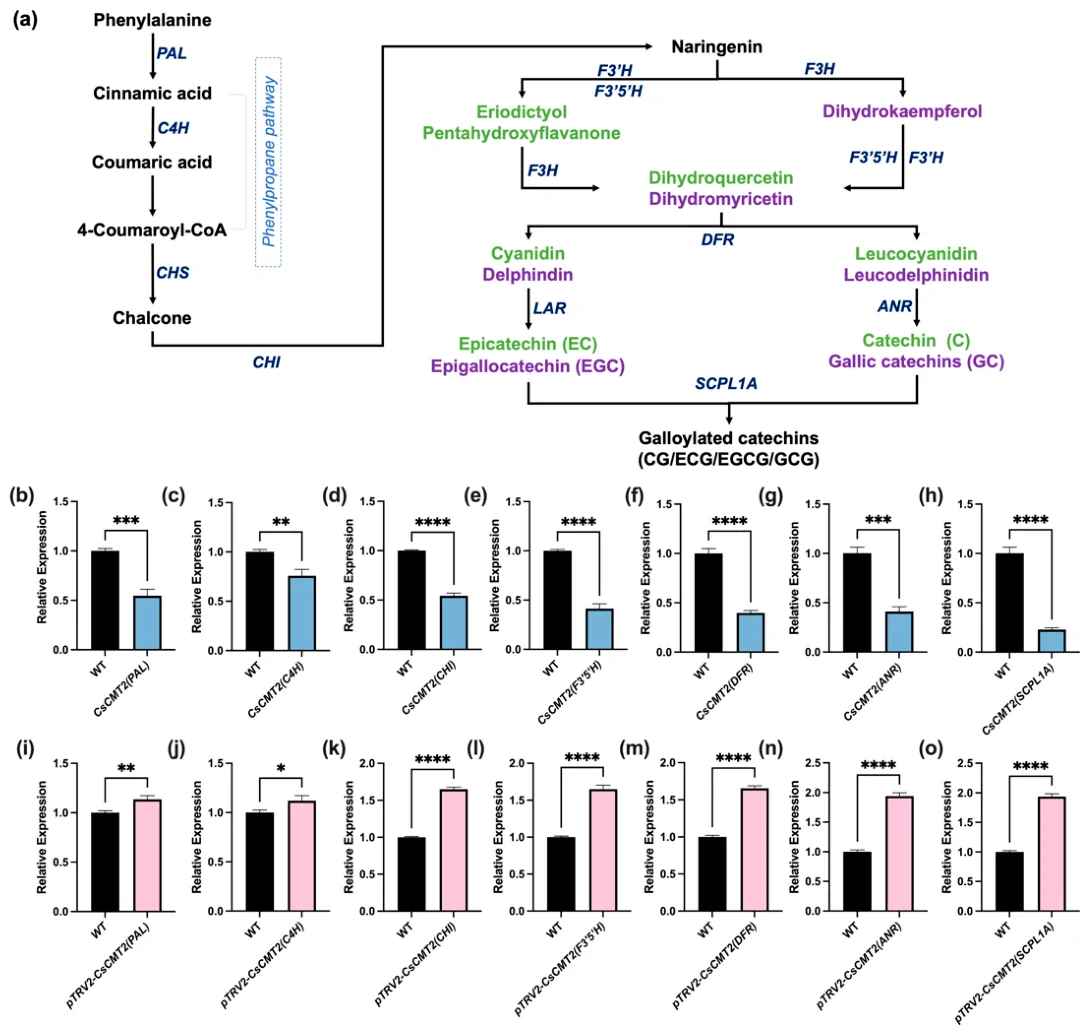
Effect of *CsCMT2* overexpression and silencing on the expression of catechin biosynthetic genes in *Camellia sinensis* leaves. (**a**) Schematic diagram of the catechin biosynthetic pathway. (**b**–**h**) Relative transcript levels of *CsPAL*, *CsC4H*, *CsCHI*, *CsF3′5′H*, *CsDFR*, *CsANR* and *CsSCPL1A* in *CsCMT2*- overexpression leaves. (**i**–**o**) Relative transcript abundance of the same seven genes in *CsCMT2*-silenced (pTRV2-*CsCMT2*). Data are presented as mean ± SD (*n* = 3). Asterisks indicate significance versus WT (one-way ANOVA with Tukey’s HSD; * *p* < 0.05, ** *p*< 0.01, *** *p* < 0.001, **** *p* < 0.0001).

The expression of genes encoding enzymes of the catechin biosynthetic pathway (Figure 4a) responded reciprocally to *CsCMT2* manipulation. Overexpression of *CsCMT2* significantly downregulated all seven assayed structural genes spanning the pathway, *CsPAL*, *CsC4H*, *CsCHI*, *CsF3′5′H*, *CsDFR*, *CsANR* and *CsSCPL1A* (Figure 4b–h), whereas virus-induced gene silencing (VIGS) of *CsCMT2* produced the converse response, significantly upregulating the identical set of genes (Figure 4i–o). Every step assayed responded, from the entry into general phenylpropanoid metabolism through to the terminal galloylation reaction, with the strongest changes in *CsSCPL1A*, *CsDFR*, *CsF3′5′H,* and *CsANR* and the weakest in *CsPAL* and *CsC4H*.

The transcript and metabolite data were highly concordant, with *CsCMT2* overexpression reducing both the pathway transcripts and all six catechins, while silencing raised both. The increase after silencing was smaller than the decrease after overexpression, which is expected given that VIGS reduced endogenous *CsCMT2* transcript only partially while overexpression produced a large excess. CsCMT2 therefore appears to negatively affect catechin biosynthesis, accompanied by reduced expression of pathway genes.

## 4. Discussion

Tea quality and pharmacological value of tea depend on understanding how its key functional bioactive quality components are formed in the tea plant (*C. sinensis*). Catechins are central to both attributes, because they are major contributors to the astringency and bitterness characteristic quality of tea infusions and to their antioxidant and other health-related activities [8,10,15]. Previous multi-omics studies have associated DNA methylation with the biosynthesis of catechins and other flavor compounds in tea [49,50,61] but the contribution of individual DNA methyltransferase genes to these processes has remained unclear. Although CMT genes have been identified in tea, previous analyses focused mainly on their responses to abiotic stress and withering [48]. In this study, we further characterized the structural features and expression patterns of the tea *CMT* genes and identified *CsCMT2* as the member with the most pronounced cultivar-dependent expression under *C. gloeosporioides* infection. Reciprocal overexpression and silencing then showed that *CsCMT2* negatively affects catechin accumulation without significantly affecting caffeine accumulation. These results provide functional evidence that a chromomethylase gene influences catechin accumulation and tea quality-related metabolism.

The tea CMT family comprises four genes representing the three plant CMT subfamilies [42]. CsCMT3a and CsCMT3b are nearly identical tandem duplicates located 43 kb apart on chromosome 7, and their almost complete sequence is consistent with a duplication event. This high similarity also explains why their transcripts could not be distinguished and were quantified together as CsCMT3. In contrast, CsCMT2 is structurally distinct from the other members. It is the most divergent protein and the only basic member of the family, and it is encoded by the largest locus with the fewest exons. It also has a markedly shortened N-terminal region in which the BAH domain was not detected, while its chromodomain and catalytic domain remain intact.

The expression patterns of the *CsCMT* genes are closely aligned with the tissues that determine tea quality. All three genes demonstrated elevated expression levels in buds and young leaves, while their expression levels declined in mature and old leaves. These tender tissues are the raw material harvested for tea production and are also where total catechins and galloylated catechins such as EGCG accumulate most strongly, with their contents decreasing as leaves mature [23]. The *CsCMT* genes are therefore expressed most strongly in the tissues in which the catechin profile of tea is established. Within the family, three lines of evidence pointed specifically to *CsCMT2*. First, *CsCMT2* is the only tea member of the CMT2 subfamily, which mainly mediates non-CG methylation, specifically in the CHH context [44,45]. In tea leaves, the effects of DNA methylation on catechin biosynthetic genes depend on the sequence context, and changes in the methylation and expression of genes such as *CsSCPL1A* and *CsLAR* are consistent with changes in catechin content [61]. Second, *CsCMT2* showed the most pronounced expression variation among the eight cultivars infected with *C. gloeosporioides*. Anthracnose reduces both the yield and the quality of tea [51], and catechin accumulation is part of the defense response of tea plants against this pathogen [52]. Because DNA methylation can be dynamically remodeled during plant responses to pathogens [62], the strong genotype-dependent expression of *CsCMT2* under infection suggested that *CsCMT2* may contribute to the regulation of catechin metabolism. Third, *CsCMT2* has no close paralog in tea, so its function can be examined without the redundancy associated with the tandemly duplicated *CsCMT3a* and *CsCMT3b*. Together, these considerations led to the hypothesis that *CsCMT2* is involved in regulating catechin biosynthesis, which the functional assays then supported. In other medicinal plants such as *Epimedium* species, flavonoid accumulation is associated with regulatory hubs involving MYB transcription factors (TFs) and glycosyltransferases [63]; *CsCMT2* may therefore represent an additional epigenetic layer within such flavonoid regulatory networks.

The overexpression and silencing assays yielded consistent and reciprocal results. Overexpression of *CsCMT2* decreased all six measured catechins and downregulated all seven assayed biosynthetic genes, from the phenylpropanoid entry steps catalyzed by *CsPAL* and *CsC4H* to the galloylation step catalyzed by *CsSCPL1A*, whereas silencing of *CsCMT2* had the opposite effects. Two observations support the specificity of this regulation. First, caffeine, which is synthesized through an independent purine alkaloid pathway, was not significantly affected by either manipulation. Second, the empty-vector and pTRV2 controls did not significantly differ from wild types (WT), vividly illustrating that the changes were not caused by the infiltration procedure or by TRV infection. The smaller increase in catechin content after silencing than the decrease after overexpression is consistent with the partial reduction in *CsCMT2* transcripts achieved by VIGS, compared with the strong transcript increase produced by overexpression. Because the transcript levels of catechin biosynthetic genes are closely associated with catechin accumulation in tea [25,26], the coordinated downregulation of these genes is consistent with the reduced catechin content after *CsCMT2* overexpression. In both assays, *CsSCPL1A*, *CsANR*, *CsDFR*, and *CsF3′5′H* responded more strongly than *CsPAL* and *CsC4H*, suggesting that CsCMT2 has a stronger effect on the later steps of the pathway, which determine the composition of the catechin pool. Given that CMT2-type enzymes mainly mediate non-CG methylation [44,45], and that the methylation state of catechin biosynthetic genes has been linked with their expression in tea [61]. It is possible that *CsCMT2* affects these genes through DNA methylation. However, methylation levels were not determined in this study, and this possibility remains to be tested. In addition, all functional assays were performed in the cultivar ‘Shuchazao’; because tea cultivars differ in both catechin composition and *CsCMT2* expression (Figure 2e), the magnitude of the metabolic changes reported here may vary among genotypes, and validation in additional cultivars would be needed to assess the generality of these effects.

Although flavor attributes were not assessed directly in this study, the observed changes in catechin composition have potential implications for tea flavor quality. Galloylated catechins, particularly EGCG, are major contributors to the astringency of tea infusions [10] and to the antioxidant and pharmacological activities of tea [13,15,16]. EGCG accounted for most of the absolute catechin loss after *CsCMT2* overexpression. In addition, *CsSCPL1A*, which catalyzes catechin galloylation, was the most strongly downregulated gene. This suggests that *CsCMT2* influences not only total catechin content but also the galloylated catechin fraction, which is closely associated with both astringency and health-promoting activity. Because caffeine content was not significantly affected, *CsCMT2* may allow the catechin content of tea leaves, and thus their astringency and total polyphenol content, to be adjusted largely independently of caffeine. These observations are consistent with recent studies on catechin regulation in tea plants. Integrated transcriptomic and metabolomic analyses of tender tea leaves have identified MYB and Dof transcription factors as potential regulators of cultivar-dependent flavonoid accumulation, including that of catechin and epicatechin [64], suggesting that catechin composition in the harvested tissues is shaped by multiple transcriptional regulators. Galloylated catechin biosynthesis further depends on gallic acid supply by shikimate dehydrogenases [65] and on galloyl transfer by serine carboxypeptidase-like acyltransferases [66], both of which are key determinants of galloylated catechin levels in Camellia sinensis plants. In the present study, *CsCMT2* overexpression strongly suppressed *CsSCPL1A* expression, suggesting that variation in chromomethylase expression may be an additional epigenetic factor regulating galloylated catechin biosynthesis.

This property offers opportunities for the improvement of tea germplasm and for the development of raw materials suited to different tea products. Tea cultivars differ considerably in catechin content and composition, and these differences determine their suitability for particular tea types [8,17]. Based on the functional assays, lower *CsCMT2* activity would be expected to favor catechin-rich raw materials suited to health-promoting properties of tea products and catechin extraction. Higher *CsCMT2* activity would instead be expected to favor lower catechin levels and a less astringent, mellower taste, similar to that of teas grown under conditions that reduce catechin accumulation [22]. Because catechin content also varies with season and growing conditions [26,50], *CsCMT2* may be one of the genetic factors through which such conditions influence leaf quality. Anthracnose, a predominant fungal disease in tea plantations, is also a common constraint in tea production that reduces both the yield and quality of harvested tea leaves [51]. The pronounced cultivar-dependent expression of *CsCMT2* under *C. gloeosporioides* infection therefore suggests that this gene may be relevant to how different cultivars maintain their catechin profiles, and thus their flavor quality and health value, under disease pressure. Infection phenotypes were not assessed in the present study, and the expression data under *C. gloeosporioides* infection were used only to identify candidate *CsCMT* members for functional analysis; *CsCMT2* is therefore not characterized here as a pathogen-resistance gene. Furthermore, the catechin profile of fresh leaves forms the basis of the flavor and health value of processed tea, and DNA methylation-associated regulation of flavor metabolism has been reported during tea processing [49]. *CsCMT2* therefore provides a promising target for linking germplasm improvement with the augmented quality of the final tea product.

Overall, this study focused on the role of *CsCMT2* in regulating catechin biosynthesis and tea quality-related metabolism. The reciprocal overexpression and silencing assays, the concomitant changes in catechin contents and pathway gene expression, and the unchanged caffeine levels consistently support *CsCMT2* as a candidate negative regulator of catechin accumulation in tea plants. These findings extend the current understanding of how chromomethylase genes contribute to tea quality formation and provide a basis for applying CsCMT2 in the improvement of tea flavor quality and health value.

## 5. Conclusions

In this study, we characterized four chromomethylase genes in *Camellia sinensis*, *CsCMT1*, *CsCMT2*, *CsCMT3a*, and *CsCMT3b*, which represent the three plant CMT subfamilies. Among them, *CsCMT2*, the only tea member of the CMT2 subfamily, is structurally distinct from the other members. The *CsCMT* genes were preferentially expressed in buds and young leaves, the tissues harvested for tea production, and CsCMT2 showed the most pronounced cultivar-dependent expression under Anthracnose (*Colletotrichum gloeosporioides*) infection. In transient assays in the cultivar ‘Shuchazao’, overexpression of *CsCMT2* decreased the contents of all six measured catechins, with EGCG accounting for most of the reduction, and downregulated seven catechin biosynthetic genes, most strongly the galloylation gene *CsSCPL1A*. Conversely, silencing of *CsCMT2* increased total catechin content and upregulated these genes, whereas caffeine content was not significantly affected by either manipulation. These results identify *CsCMT2* as a candidate negative regulator of catechin biosynthesis and tea quality-related metabolism. Because *CsCMT2* influences catechin accumulation largely independently of caffeine, it represents a promising target for tailoring astringency, flavor quality, and catechin composition in tea germplasm improvement.

## Data Availability

The original contributions presented in this study are included in the article/Supplementary Material. Further inquiries can be directed to the corresponding authors.

## Supplementary Materials

The following supporting information can be downloaded at: https://www.mdpi.com/article/10.3390/foods15203618/s1, Figure S1: Conserved domains and motifs of the CsCMT family; Figure S2: Leaf phenotypes after transient CsCMT2 overexpression and VIGS; Figure S3: Representative HPLC chromatogram; Table S1: Summary information of the four *CsCMT* genes identified in *C. sinensis* with genes, gene ID, CDS sequences, protein (aa), MW (kDa), pI, Instab.index, aliph.index, GRAVY, and localization; Table S2: Pairwise amino acid identity (%) among the four CsCMT proteins; Table S3: Genomic coordinates and structure of the *CsCMT* genes; Table S4: *Cis*-acting elements predicted in CsCMT promoters; Table S5: Primers used in this study; Table S6: Total catechin content, relative composition of the catechin pool, and fold change in individual catechins; Table S7: Sequence comparison and off-target analysis of CsCMT2 VIGS fragment against the other *CsCMT* genes.
