# Supplementary material for: The Chromomethylase CsCMT2 Negatively Affects Catechin Accumulation in Tea Plants
Source: Foods. 2026 Oct 11;15(20):3618. doi: 10.3390/foods15203618 (PMC13703429; doi:10.3390/foods15203618)

**Supplementary Table S1.** Summary information of the four *CsCMT* genes identified in *C. sinensis* with genes, gene ID, CDS sequences, protein (aa), MW (kDa), *pI*, Instab.index, aliph.index, GRAVY, and localization.

| **Gene** | **Gene ID** | **CDS (bp)** | **Protein (aa)** | **MW (kDa)** | **pI** | **Instab. index** | **Aliph. index** | **GRAVY** | **Localization** |
|---|---|---|---|---|---|---|---|---|---|
| *CsCMT1* | CSS0013017 | 2,343 | 780 | 87.60 | 6.01 | 36.81 | 83.09 | −0.280 | Nucleus |
| *CsCMT2* | CSS0003392 | 1,875 | 624 | 70.19 | 8.29 | 33.80 | 80.42 | −0.337 | Nucleus |
| *CsCMT3a* | CSS0038763 | 2,538 | 845 | 95.55 | 4.95 | 41.18 | 77.94 | −0.575 | Nucleus |
| *CsCMT3b* | CSS0034027 | 2,475 | 824 | 93.41 | 4.95 | 42.11 | 79.45 | −0.542 | Nucleus |

**Supplementary Table S2.** Pairwise amino-acid identity (%) among the four CsCMTs proteins (CsCMT1, CsCMT2, CsCMT3a, and CsCMT3b)

| | ***CsCMT1*** | ***CsCMT2*** | ***CsCMT3a*** | ***CsCMT3b*** |
|---|---|---|---|---|
| **CsCMT1** | 100.0 | 46.9 | 55.3 | 56.3 |
| **CsCMT2** | 46.9 | 100.0 | 45.5 | 46.4 |
| **CsCMT3a** | 55.3 | 45.5 | 100.0 | 97.5 |
| **CsCMT3b** | 56.3 | 46.4 | 97.5 | 100.0 |

**Supplementary Table S3.** Genomic coordinates and structure of the *CsCMT* genes, where genes, gene ID, Chr., position (bp), strands, span (bp), and protein (aa).

| **Gene** | **Gene ID** | **Chr.** | **Position (bp)** | **Strand** | **Span (bp)** | **CDS (bp)** | **Protein (aa)** |
|---|---|---|---|---|---|---|---|
| *CsCMT1* | CSS0013017 | Chr13 | 97,580,578–97,589,595 | − | 9,018 | 2,343 | 780 |
| *CsCMT2* | CSS0003392 | Chr07 | 184,579,959–184,605,157 | − | 25,199 | 1,875 | 624 |
| *CsCMT3a* | CSS0038763 | Chr07 | 41,120,819–41,129,087 | + | 8,269 | 2,538 | 845 |
| *CsCMT3b* | CSS0034027 | Chr07 | 41,070,029–41,077,865 | + | 7,837 | 2,475 | 824 |

**Supplementary Table S4.** *Cis*-acting elements predicted in *CsCMT* promoters. Twenty cis-elements grouped into light, hormone, stress, and growth development related categories across the four CsCMT promoters.

| **Functional category** | ***cis-element*** | ***CsCMT1* (CSS0013017)** | ***CsCMT2* (CSS0003392)** | ***CsCMT3a* (CSS0038763)** | ***CsCMT3b* (CSS0034027)** |
|---|---|---|---|---|---|
| | G-box | 8 | 5 | 7 | 7 |
| | Box 4 | 3 | 2 | 4 | 4 |
| **Light-responsive** | GT1-motif | 3 | 1 | 3 | 3 |
| | ACE | 2 | 1 | 2 | 2 |
| | AE-box | 2 | 1 | 2 | 2 |
| | ABRE | 4 | 2 | 5 | 5 |
| | CGTCA-motif | 2 | 1 | 3 | 3 |
| **Hormone-responsive** | TGACG-motif | 2 | 1 | 2 | 2 |
| | TCA-element | 3 | 1 | 2 | 2 |
| | TGA-element | 3 | 2 | 3 | 3 |
| | P-box | 2 | 1 | 2 | 2 |
| | ARE | 5 | 3 | 4 | 4 |
| | MBS | 2 | 2 | 1 | 1 |
| **Stress-responsive** | LTR | 3 | 1 | 3 | 3 |
| | TC-rich repeats | 6 | 3 | 5 | 5 |
| | WUN-motif | 2 | 1 | 2 | 2 |
| | CAT-box | 2 | 1 | 1 | 1 |
| **Growth & development** | O2-site | 2 | 1 | 1 | 1 |
| | HD-Zip1 | 1 | 0 | 1 | 1 |
| | circadian | 3 | 1 | 2 | 2 |

**Supplementary Table S5**. The primer sequences used in this study

| Primer | Sequence 5'-3 |
|---|---|
| *CsCMT2-F* | ATGAAATATTCAGTGGACTATTCAA |
| *CsCMT2-R* | AATAGGATGACGATTGCAACA |
| *arm-XbaI-CsCMT2-F* | GAGAACACGGGGGACTCTAGAATGAAATATTCAGTGGACTATTCAA |
| *arm-XhoI-CsCMT2-R* | GGTAGTCGACGGTACCTCGAGAATAGGATGACGATTGCAACA |
| *pTRV2-CsCMT2-F* | AGTAAGGTTACCGAATTC ACTACCAAGCAAGGCTAGGAAT |
| *pTRV2-CsCMT2-R* | GAGCTCGGTACCGGATCC TACAGGAGGAAGGTCACAGATG |
| *RT q-PCR CsCMT1-F* | ACCTTTGGTCCCAGATTA |
| *RT q-PCR CsCMT1-R* | CAACAGCATTCCCTACCT |
| *RT q-PCR CsCMT2-F* | TATGATGGGCTCTGCTTCC |
| *RT q-PCR CsCMT2-R* | AATTTGCTCCCTTTCTTTCG |
| *RT q-PCR CsCMT3-F* | TATGGTGGGATGAGACGC |
| *RT q-PCR CsCMT3-R* | AGCAACTGGAACGGCAAC |
| *RT q-PCR CsPAL-F* | CGTGTTGTCAGAAGTTTTGT |
| *RT q-PCR CsPAL-R* | ATGGGTCCATTTCATGTAGT |
| *RT q-PCR CsC4H-F* | CGATTTTATTCCGATTTTGA |
| *RT q-PCR CsC4H-R* | TGATCTCTCCCTTCTGTTGA |
| *RT q-PCR CsCHI-F* | TGGATGAGATGAGGAAGAAG |
| *RT q-PCR CsCHI-R* | GATTAACCCAACCAGGAAGT |
| *RT q-PCR CsF3H-F* | AAGTTGCTGGAGGTGTTGTC |
| *RT q-PCR CsF3H-R* | ATCCGTGTGTCGCTTGAGTC |
| *RT q-PCR CsDFR-F* | ATGGAAGTAGTGGAAGTGAA |
| *RT q-PCR CsDFR-R* | CAAGCTGAGGTGTGTGGTAG |
| *RT q-PCR CsANR-F* | ATTCTGCGAAGCCGCCCACT |
| *RT q-PCR CsANR-R* | CATACCTTTCAACCCATTTA |
| *RT q-PCR CsSCPL 1A-F* | ATTTCATTCTCTCACCTCCC |
| *RT q-PCR CsSCPL 1A-R* | GTTCTTGTTGCATCTCTTCC |

**Supplementary Table S6.** Total catechin content, relative composition of the catechin pool, and fold change of individual catechins

| Genotype | Total catechins (mg/g DW) | Relative composition (% of total catechins) | | | | | | Fold change | | | | | |
|---|---|---|---|---|---|---|---|---|---|---|---|---|---|
| | | EGCG | GCG | EGC | GC | EC | C | EGCG | GCG | EGC | GC | EC | C |
| ***Overexpression*** | | | | | | | | | | | | | |
| WT | 103.67 ± 2.24 | 63,9 | 5,4 | 18,2 | 3,8 | 6,7 | 2,1 | 1,01 | 0,99 | 1,01 | 1,04 | 1,03 | 1,04 |
| Empty vector | 102.18 ± 2.03 | 64,0 | 5,5 | 18,2 | 3,7 | 6,6 | 2,0 | 1,00 | 1,00 | 1,00 | 1,00 | 1,00 | 1,00 |
| *CsCMT2-1* | 39.93 ± 1.16 | 65,7 | 6,2 | 13,5 | 4,8 | 7,4 | 2,4 | 0,40 | 0,44 | 0,29 | 0,51 | 0,44 | 0,47 |
| *CsCMT2-2* | 44.93 ± 0.80 | 66,4 | 6,0 | 13,6 | 4,6 | 6,9 | 2,6 | 0,46 | 0,48 | 0,33 | 0,55 | 0,46 | 0,56 |
| *CsCMT2-3* | 38.30 ± 0.30 | 66,1 | 5,5 | 13,8 | 4,9 | 7,4 | 2,4 | 0,39 | 0,37 | 0,29 | 0,50 | 0,42 | 0,44 |
| ***VIGS*** | | | | | | | | | | | | | |
| WT | 103.67 ± 2.24 | 63,9 | 5,4 | 18,2 | 3,8 | 6,7 | 2,1 | 1,01 | 1,05 | 1,01 | 1,03 | 1,01 | 1,06 |
| pTRV2 | 102.02 ± 2.25 | 64,1 | 5,3 | 18,2 | 3,7 | 6,7 | 2,0 | 1,00 | 1,00 | 1,00 | 1,00 | 1,00 | 1,00 |
| pTRV2-*CsCMT2-1* | 112.40 ± 2.10 | 63,3 | 5,4 | 18,5 | 3,8 | 6,8 | 2,2 | 1,09 | 1,14 | 1,12 | 1,12 | 1,11 | 1,22 |
| pTRV2-*CsCMT2-2* | 112.98 ± 1.92 | 63,2 | 5,4 | 18,5 | 3,8 | 6,8 | 2,2 | 1,09 | 1,15 | 1,13 | 1,13 | 1,12 | 1,24 |
| pTRV2-*CsCMT2-3* | 112.70 ± 2.51 | 63,3 | 5,4 | 18,5 | 3,8 | 6,8 | 2,2 | 1,09 | 1,14 | 1,12 | 1,13 | 1,12 | 1,24 |

**Supplementary Table S7.** Nucleotide sequence and off-target analysis of the 254-bp CsCMT2 fragment used for virus-induced gene silencing (VIGS).

### CsCMT2 VIGS fragment (254 bp; 5′–3′):

```
ACTACCAAGCAAGGCTAGGAATTATGGCTGCTGGGTGTTATGGTCTTCCTCAATTCCGTT
TGCGTGTTTTCTTGTGGGGCGCTCATTCCCATGAGAAATTACCTCAGTTTCCACTTCCCA
CACATGATGTGGTTTTGAAGTATGGGGCTCCAAGGGAGTTTGAGCGTTGTGTTGTTGCTT
ATGATGAAGGCCAGTCTCGAGAGTTGGAAAAAGCTCTTGTTCTTAGTGATGCCATCTGTG
ACCTTCCTCCTGTA
```

| Gene | Gene ID | Identity with fragment (%) | Longest contiguous identity (nt) | Number of 21-nt exact matches |
|---|---|---|---|---|
| *CsCMT1* | CSS0013017 | 64.3 | 11 | 0 |
| *CsCMT3a* | CSS0038763 | 67.6 | 18 | 0 |
| *CsCMT3b* | CSS0034027 | 67.6 | 18 | 0 |

**Supplementary Figure S1.** Conserved domains and motifs of the CsCMT family. (a) MEME motif distribution and (b) Domain architecture. Each protein retains the catalytic DNA methyltransferase domain and chromo-domain; the N-terminal BAH domain was not detected in the current CsCMT2 protein model.

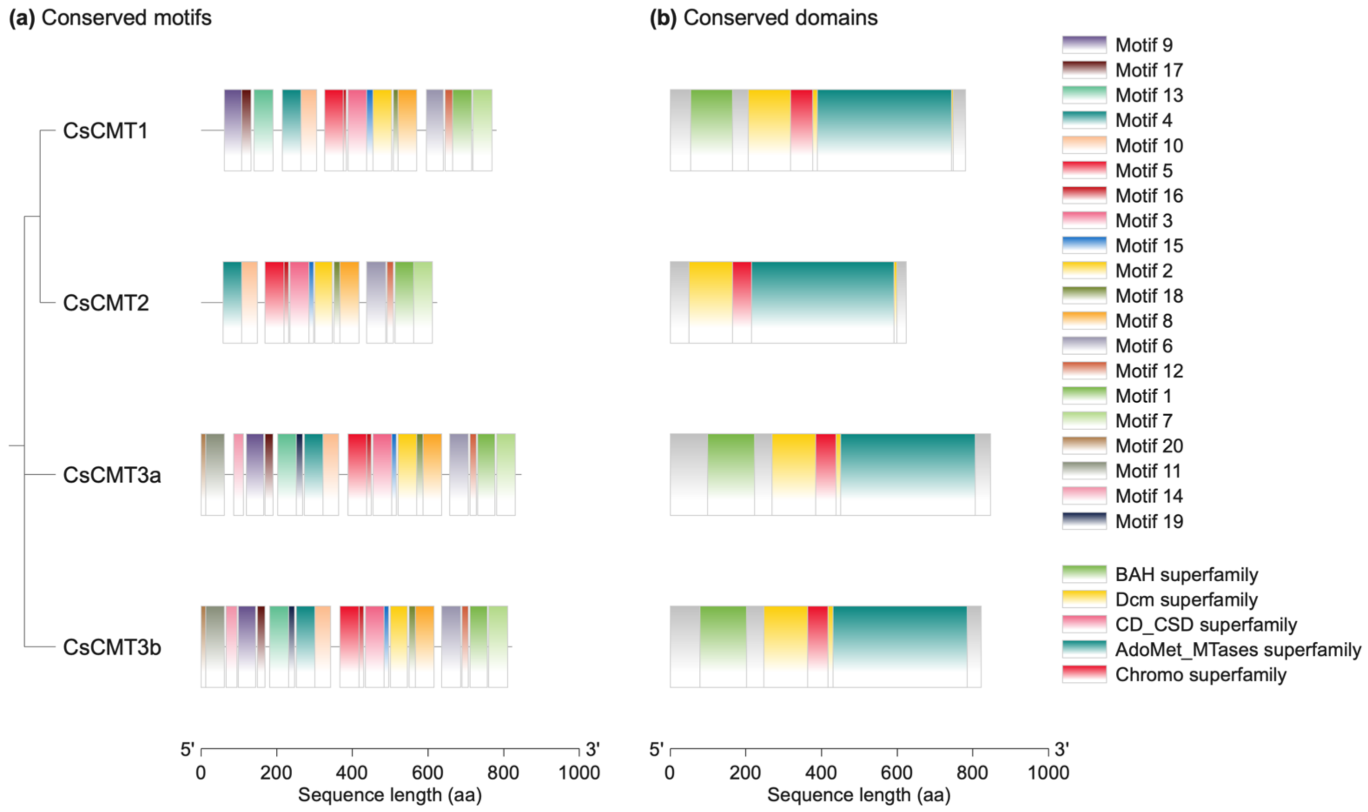

**Supplementary Figure S2.** Leaf phenotypes. (a) *CsCMT2* overexpression and (b) *CsCMT2*-silenced (VIGS) in *C.sinensis*. Scale bar : 1 cm.

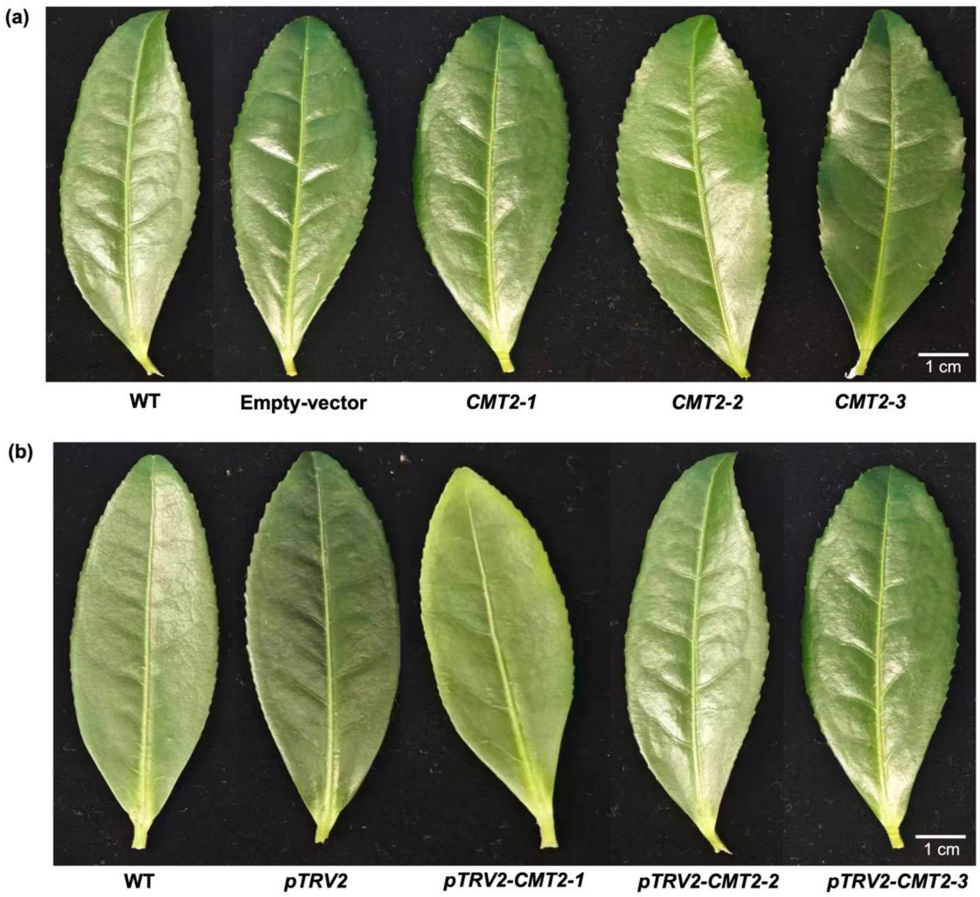

**Supplementary Figure S3.** Representative HPLC chromatogram. GC, gallocatechin; EGC, epigallocatechin; C, catechin; CAF, caffeine; EGCG, epigallocatechin gallate; EC, epicatechin; GCG, gallocatechin gallate. Numbers beside each label are retention times (min). Unlabelled peaks correspond to the solvent front and non-target compounds.

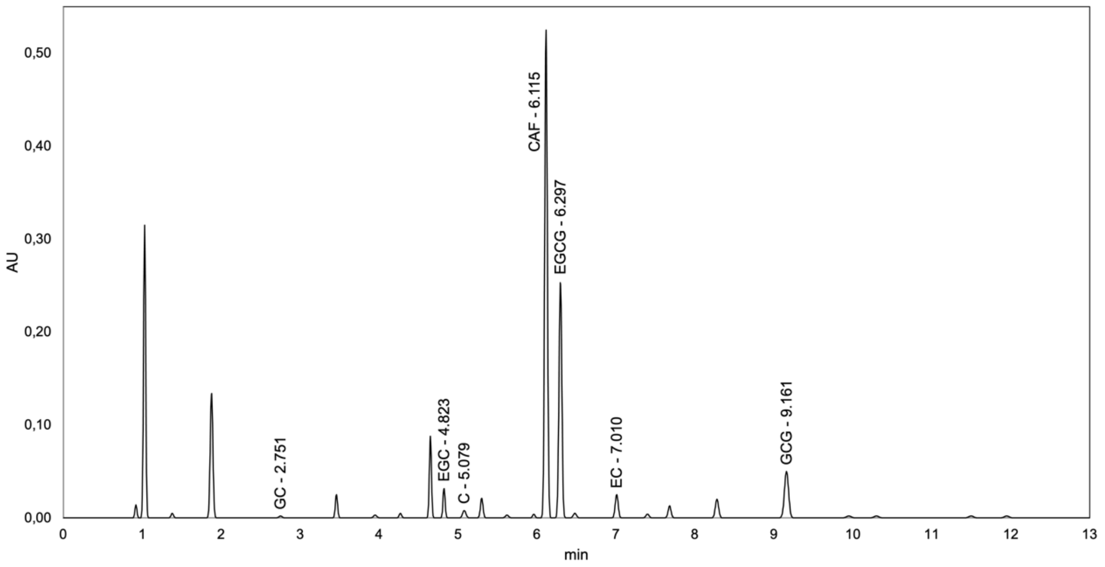

Supplement: Supplementary file 1 [file foods-15-03618-s001.zip › foods-4604527-supplementary.pdf]
